# Assessment of severity and frequency of self-reported hypoglycemia on quality of life in patients with type 2 diabetes treated with oral antihyperglycemic agents: A survey study

**DOI:** 10.1186/1756-0500-4-251

**Published:** 2011-07-21

**Authors:** Elizabeth Marrett, Larry Radican, Michael J Davies, Qiaoyi Zhang

**Affiliations:** 1 Global Health Outcomes, Merck Sharp & Dohme, Corp., Whitehouse Station, NJ, USA; 2 Department of Epidemiology, University of Medicine and Dentistry of New Jersey - School of Public Health, Piscataway, NJ, USA

## Abstract

**Background:**

Some oral antihyperglycemic agents may increase risk of hypoglycemia and thereby reduce patient quality of life. Our objective was to assess the impact of the severity and frequency of self-reported hypoglycemia on health-related quality of life (HRQoL) among patients with type 2 diabetes treated with oral antihyperglycemic agents.

**Findings:**

A follow-up survey was conducted in participants with self-reported type 2 diabetes treated with oral antihyperglycemic agents from the US National Health and Wellness Survey 2007. Data were collected on the severity and frequency of hypoglycemic episodes in the 6 months prior to the survey, with severity defined as mild (no interruption of activities), moderate (some interruption of activities), severe (needed assistance of others), or very severe (needed medical attention). HRQoL was assessed using the EuroQol-5D Questionnaire (EQ-5D) US weighted summary score (utility) and Worry subscale of the Hypoglycemia Fear Survey (HFS). Of the participants who completed the survey (N = 1,984), mean age was 58 years, 57% were male, 72% reported an HbA_1c _<7.0%, and 50% reported treatment with a sulfonylurea-containing regimen. Hypoglycemic episodes were reported by 63% of patients (46% mild, 37% moderate, 13% severe and 4% very severe). For patients reporting hypoglycemia, mean utility score was significantly lower (0.78 versus 0.86, p < 0.0001) and mean HFS score was significantly higher (17.5 versus 6.2, p < 0.0001) compared to patients not reporting hypoglycemia. Differences in mean scores between those with and without hypoglycemia increased with the level of severity (mild, moderate, severe, very severe) for utility (0.03, 0.09, 0.18, 0.23) and HFS (6.1, 13.9, 20.1, 25.6), respectively. After adjusting for age, gender, weight gain, HbA_1c_, microvascular complications, and selected cardiovascular conditions, the utility decrement was 0.045 (by level of severity: 0.009, 0.055, 0.131, 0.208), and the HFS increase was 9.6 (by severity: 5.3, 12.4, 17.6, 23.2). HRQoL further decreased with greater frequency of hypoglycemic episodes.

**Conclusions:**

Self-reported hypoglycemia is independently associated with lower HRQoL, and the magnitude of this reduction increases with both severity and frequency of episodes in patients with type 2 diabetes treated with oral antihyperglycemic agents.

## Background

Lowering blood glucose levels with the use of antihyperglycemic agents has been shown to reduce the risk of diabetic complications [1]. However, achievement of adequate glycemic control is low for many patients with type 2 diabetes [2]. One reason for this may be that long-term maintenance of glycemic control is often a lower priority than the more immediate risk of hypoglycemia [3]. This treatment limitation, induced by some glucose-lowering medications, may be a major barrier to successful diabetes care [4,5].

The primary cause of hypoglycemia among patients with type 2 diabetes is antihyperglycemic medications - in particular, those which raise insulin levels independently of blood glucose, such as sulfonylureas and insulin [6]. Hypoglycemic episodes can occur with differing severity, from the relatively minor (sweating, hunger, and anxiety) to the very severe (behavioral changes, cognitive impairment, seizures, and coma). The clinical impact of severe hypoglycemia is substantial in terms of morbidity, mortality, and quality of life [6]. The well-being of patients may be affected both directly as a result of hypoglycemia and indirectly due to fear of recurring episodes [3]. Furthermore, even mild hypoglycemia may be enough to effect clinical management of diabetes if patient fear compromises their willingness to take medications as directed [6-8].

Recently, we found that diabetes treatment-related side effects (i.e., hypoglycemia and weight gain) were associated with decreased treatment satisfaction and health-related quality of life (HRQoL) in US patients treated with oral antihyperglycemic agents [9]. The purpose of the present analysis was to examine the association between hypoglycemia and HRQoL based upon the combination of severity and frequency of hypoglycemic episodes to better understand the impact of this treatment-related side effect in US patients with type 2 diabetes treated with oral antihyperglycemic therapy.

## Methods

### Survey Sample and Administration

As previously described by Marrett et al. [9], a representative sample of the US population was identified through the 2007 National Health and Wellness Survey (NHWS), an annual cross-sectional, internet-based survey of demographics, disease status, healthcare attitudes, behaviors, and outcomes of adults aged 18 and older. The NHWS sample was stratified by age, gender, and race/ethnicity to reflect the demographic composition of the US adult population and verified against national health statistics. NHWS participants (N = 63,012) were recruited through an internet-based consumer panel, and each completed the survey at 1 of 3 different time points in 2007 - February-March, April or August.

Based on the results from the original NHWS sample, 6,349 respondents had self-reported a diagnosis of type 2 diabetes. These individuals were randomly re-contacted in November 2007 and asked if they would be willing to participate in a second internet-based survey related to their diabetes. The respondents were also screened to include only those who reported being treated with one or more oral antihyperglycemic agents any time during the previous 6 months. Excluded were those who reported insulin use during the same time period. Data collection was conducted until 2,000 follow-up surveys were completed. An Institutional Review Board (Essex IRB, Lebanon, NJ) reviewed and approved the protocol and patients provided informed consent.

### Data Collection

Patient demographics and disease characteristics were collected from the NHWS. These included age, gender, height, weight, and duration of diabetes. Additional data collected were history of potential microvascular complications (macular edema, kidney disease, foot or leg ulcers, and neuropathic pain,) and selected cardiovascular conditions (angina, heart attack, stroke, peripheral vascular disease, and congestive heart failure). At the second survey patients were asked to provide information on recent HbA_1c _measurements, current use of oral antihyperglycemic agents, and medication side effects (weight gain in 12 months and symptoms of hypoglycemia 6 months prior to the second survey).

In order to quantify the frequency and severity of hypoglycemia, patients were asked to read a list of hypoglycemic symptoms and record the frequency of such symptoms by level of severity. Consistent with the recommendations of the American Diabetes Association Workgroup on Hypoglycemia [10], hypoglycemic severity was categorized as 1) mild (little or no interruption of activities and no assistance needed to manage symptoms); 2) moderate (some interruption of activities and no assistance needed to manage symptoms); 3) severe (needed the assistance of others to manage symptoms). A fourth category of very severe hypoglycemia, was added to capture episodes that required medical assistance. Patients reported the frequency of hypoglycemia for each level of hypoglycemic symptom severity (mild, moderate, severe) during the 6 months preceding the survey. The frequency data were collected as 1-2 episodes, 3-6 episodes, >one per month, >one per week, or daily. Very severe hypoglycemic symptoms were recorded as the absolute number of episodes experienced over the last 6 months.

Two additional questionnaires were administered during the survey. HRQoL was quantified using the non-disease specific EuroQol-5D Questionnaire (EQ-5D; utility) [11]. For the purposes of this study, the unweighted summary scores were transformed to US preference-weighted index scores (-0.038 to 1.0) [12]. Worry about hypoglycemia was measured using the Worry subscale of the Hypoglycemia Fear Survey II (HFS) [7]. The subscale is comprised of 18 questions that measure degree of patient fear in the past 6 months, and is scaled from 0 to 72 (rated as 0 - 4 [most worry] for each question).

### Statistical Analysis

Descriptive statistics summarized patient demographics, disease characteristics, medication side effects, and patient reported outcomes. If a participant reported episodes of hypoglycemia at more than one level of severity, the individual was classified according to the most severe level reported (along with the associated frequency where applicable). For the severity categories of mild, moderate, and severe, frequency of hypoglycemia (number of episodes over 6 months) was analyzed for the following groupings: 1-2 episodes, 3-6 episodes, and >6 episodes (or >1 episode per month). For very severe hypoglycemia, frequency was categorized as either 1 episode or ≥2 episodes in the 6 month period. Characteristics of patients with and without reported hypoglycemic symptoms were compared using the t-test for continuous variables and the chi-square test for categorical variables.

Multiple linear regression, adjusting for potential confounders, was used to estimate the effect of hypoglycemia on HRQoL and to derive EQ-5D and HFS decrements relative to no hypoglycemia for 1) any reported hypoglycemia, 2) level of episode severity, and 3) level of episode frequency and severity. A backwards selection technique was used to retain variables significant at p < 0.1.

## Results

### Patient Characteristics

Of the 2,008 who participated in the survey, 24 patients were excluded from further analysis due to incomplete medication information. For the remaining 1,984 patients, mean age was 58.1 years, 57% were male, and 37% reported a weight gain in the previous 12 months (Table 1). The average duration of diabetes was 7.3 years, 72% had a reported HbA_1c _of <7.0%, and 50% reported using an oral treatment regimen that included a sulfonylurea. Characteristics of patients reporting hypoglycemic symptoms in the previous 6 months (63% of patients) were compared to those not reporting symptoms (37%) (Table 1). In general, patients were more likely to have reported hypoglycemic symptoms if they were younger, had more weight gain, had microvascular complications, had selected cardiovascular conditions, or were treated with a sulfonylurea-containing regimen (all p < 0.01). For those reporting hypoglycemic symptoms, similar proportions of men and women reported symptoms, while for those not reporting symptoms, men were more likely not to report symptoms than women (p < 0.001) (Table 1).

**Table 1:** Patient characteristics by self-reported hypoglycemic symptoms

Characteristic	All Patients	With Hypoglycemic Symptoms	Without Hypoglycemic Symptoms	p-value
	N = 1984	n = 1248 (63%)	n = 736 (37%)	
Female (%)	43	47	37	<.0001^§^
Male (%)	57	53	63	
Age, years	58.1 ± 11.1	56.8 ± 11.2	60.4 ± 10.4	<.0001^‡^
Body mass index, kg/m^2^	34.5 ± 8.2	34.8 ± 8.4	34.0 ± 7.9	0.0229^‡^
Duration of diabetes, years	7.3 ± 6.4*	7.2 ± 6.2	7.5 ± 6.6	0.2737^‡^
HbA_1c _<7%^† ^(%)	72.2	71.4	73.6	0.3667^§^
Any weight gain (%)	36.9	41.5	29.1	<.0001^§^
Amount of weight gain				
<10 lbs (%)	27.9	25.9	32.7	
10-20 lbs (%)	47.5	48.7	44.9	<.0001^¶^
21-30 lbs (%)	12.3	12.6	11.7	
>30 lbs (%)	12.3	12.9	10.8	
Microvascular (%)	22.5	25.8	16.9	<.0001^§^
Macular edema (%)	4.0	4.8	2.6	0.0143^§^
Kidney disease (%)	2.5	2.9	1.8	0.1210^§^
Foot or leg ulcer (%)	3.6	4.2	2.6	0.0663^§^
Neuropathic pain (%)	17.3	20.4	12.1	<.0001^§^
Selected cardiovascular conditions (%)	19.5	21.6	15.9	0.0018^§^
Angina (%)	8.5	9.9	6.3	0.0054^§^
Heart attack (%)	8.0	8.2	7.7	0.7342^§^
Stroke (%)	4.3	4.7	3.5	0.2042^§^
Peripheral vascular disease (%)	0.96	1.0	0.82	0.6169^§^
Congestive heart failure (%)	4.3	4.7	3.7	0.2983^§^
Current antihyperglycemic agents regimen				
Treatment regimen including a sulfonylurea (%)	50	55	42	<.0001^¶^
All other treatment regimens (%)	50	45	58	

Data are presented as frequency and mean ± standard deviation.

*median 6 yrs.; 25th percentile 3 yrs.; 75th percentile 10 yrs.

^†^n = 1439

Based on ^‡ ^t-test, ^§ ^chi-square, or ^¶ ^Wald test of joint significance.

### Reported Hypoglycemic Symptoms

Of patients reporting hypoglycemic symptoms and severity level (n = 1183), 45.6% experienced mild symptoms, 37.4% moderate, 13.2% severe, and 3.8% very severe symptoms (Table 2). For those reporting the frequency of hypoglycemic episodes in the 6-month period prior to the survey (n = 1172), 60% of patients reported having 1-2 episodes of hypoglycemia, 22% had 3-6 episodes, and 14% had >6 episodes (or >1 episode per month). For very severe hypoglycemic symptoms, 2.1% experienced one episode and 1.4% ≥2 episodes over the 6 month period. The breakdown of frequency groupings by levels of severity is in Table 2.

**Table 2:** Severity and frequency of self-reported hypoglycemic symptoms

Severity level	Number of self-reported symptoms (N = 1172)	
	Category*	Frequency %
Mild	1 to 2	27.8
	3 to 6	12.1
	>1/month	5.9
Moderate	1 to 2	24.0
	3 to 6	7.4
	>1/month	6.2
Severe	1 to 2	8.2
	3 to 6	2.8
	>1/month	2.1
Very Severe	1	2.1
	≥2	1.4

* Based on number of hypoglycemic episodes in previous 6 months.

### Association between Hypoglycemic Symptoms and Utility Decrements/EuroQol-5D

**Figure 1:**
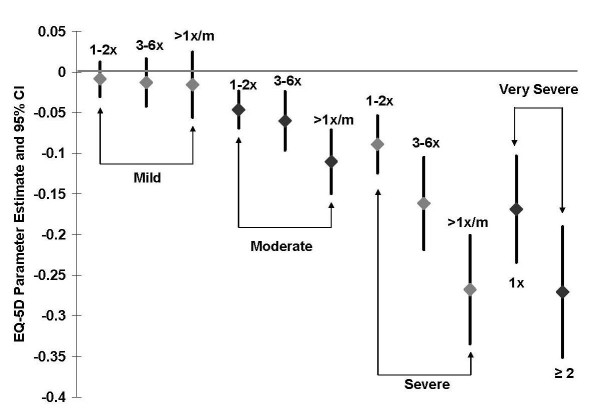
**Adjusted EQ-5D decrements by severity and frequency of hypoglycemic symptoms (based on the number of hypoglycemic episodes in previous 6 months) relative to the reference group with no hypoglycemia**.

For patients reporting any hypoglycemic symptoms, the unadjusted mean utility score was significantly lower (0.78 versus 0.86, respectively; p < .0001) compared to patients with no symptoms (Table 3). In addition, between-group differences in unadjusted mean utility score for those with and without hypoglycemic symptoms increased with symptom severity (Table 3). After adjusting for age, gender, weight gain, proportion with HbA_1c _<7% (any hypoglycemia model only), microvascular complications, and selected macrovascular conditions, the adjusted mean utility score (reference = no hypoglycemia) was 0.045 lower (p < 0.001) for those reporting any hypoglycemic symptoms and by symptom severity: mild 0.009, moderate 0.055, severe 0.131, and very severe 0.208 (Table 3). Utility scores further decreased within each severity level when frequency of hypoglycemic episodes was taken into account (Figure 1). Duration of diabetes and current medication regimen (those with a sulfonylurea vs. those without) were not significant factors (p > 0.1) and were excluded during the model backward selection.

**Table 3:** Mean EQ-5D scores and utility decrements* for presence of hypoglycemic symptoms and episode severity

Hypoglycemic Episode Level	n	Mean EQ-5D Score	Unadjusted Decrement	**Adjusted Decrement**^†^
No Hypoglycemia	736	0.86	0	0
Any Hypoglycemia	1248	0.78	-0.08	-0.05^‡^
Mild	540	0.83	-0.03	-0.01
Moderate	442	0.77	-0.09	-0.06^‡^
Severe	156	0.68	-0.18	-0.13^‡^
Very Severe	45	0.63	-0.23	-0.21^‡^

* Reference group: no hypoglycemia

^† ^Adjusted for age, gender, weight gain, proportion with HbA_1c _<7% (any hypoglycemia model only), microvascular complications and selected cardiovascular conditions.

^‡ ^Parameter estimates p < 0.0001.

### Association between Hypoglycemic Symptoms and Quality of Life Reductions/HFS

**Figure 2:**
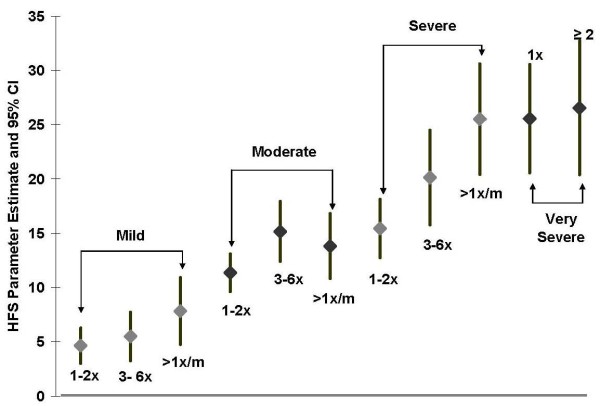
**Adjusted HFS decrements by severity and frequency of hypoglycemic symptoms (based on the number of hypoglycemic episodes in previous 6 months) relative to the reference group with no hypoglycemia**.

The unadjusted mean HFS score was significantly higher (17.5 versus 6.2, respectively; p < 0.001) for patients reporting hypoglycemic symptoms compared to patients with no symptoms, and between-group differences increased with symptom severity (Table 4). After adjusting for age, gender, weight gain, microvascular complications and selected cardiovascular conditions, the HFS score (reference = no hypoglycemia) was 9.6 points higher (p < 0.001) for patients reporting any hypoglycemic symptoms, and the between-group difference increased with symptom severity (Table 4). HFS scores further increased within each severity level when frequency of hypoglycemic episodes was taken into account (Figure 2). Duration of diabetes, proportion with HbA_1c _<7%, and current medication regimen were not significant factors (p > 0.1) and were excluded during the model backward selection.

**Table 4:** Mean HFS scores and quality of life decrements* for presence of hypoglycemic symptoms and episode severity

Hypoglycemic Episode Level	n	Mean HFS Score	Unadjusted Decrement	**Adjusted Decrement**^†^
No Hypoglycemia	736	6.2	0	0
Any Hypoglycemia	1248	17.5	11.3	9.6^‡^
Mild	540	12.3	6.1	5.3^‡^
Moderate	442	20.1	13.9	12.4^‡^
Severe	156	26.3	20.1	17.6^‡^
Very Severe	45	31.8	25.6	23.2^‡^

* Reference group: no hypoglycemia

^† ^Adjusted for age, gender, weight gain, microvascular complications and selected macrovascular conditions.

^‡ ^Parameter estimates p < 0.0001.

## Discussion

In this study of US patients with type 2 diabetes treated with oral antihyperglycemic agents, symptoms of hypoglycemia were common and reported by nearly 63% of patients, 17% of whom reported severe or very severe events. The proportion of patients reporting hypoglycemic symptoms in the present study is higher than that (16 to 39%) previously reported by US and European patients treated with oral antihyperglycemic agents [13-16]. The present results suggest that hypoglycemia may be more common for patients treated with oral antihyperglycemic agents than previously reported. It is difficult, however, to compare the incidence of hypoglycemia across studies due to different study designs and populations, medication regimens, and definitions of hypoglycemia [14-16].

Hypoglycemia was shown to be independently associated with lower patient utility, and that disutility increases with severity of hypoglycemic episode in the present study. Similar, although non-significant, results were obtained in a study of Swedish type 2 diabetic patients treated with oral antihyperglycemic agents and/or insulin. Based on the EQ-5D, Lundkvist et. al. [3] reported a utility decrement of 0.047 (p = 0.120) for patients experiencing any hypoglycemia in the preceding month, after adjusting for gender, insulin use, and hypoglycemia group (symptomatic, mild or severe). The relationship between severity of hypoglycemic episode and patient disutility in the present analysis was similar to that reported for French patients with type 2 diabetes treated with a combination of metformin and sulfonylurea. For patients reporting hypoglycemic episodes in the previous 6 months, the EQ-5D decreased by 0.07 for mild, 0.08 for moderate and 0.27 for severe/very severe hypoglycemic episodes, after adjusting for patient and disease characteristics [15].

Reported hypoglycemic symptoms were also demonstrated to be significantly associated with fear of hypoglycemia (as assessed by the HFS Worry scores), and that the magnitude of fear increases with severity of episode in the present study. Vexiau et al. [15] reported HFS scores that increased by 7.9 for any hypoglycemia, and by 5.8 for mild episodes, 11.1 for moderate, and 13.4 for severe episodes after adjusting for patient and disease characteristics. Although these decrements are smaller overall than those found in the present study, the positive linear association between severity of hypoglycemic episode and greater fear is consistent.

In general, patients who experience severe and/or frequent hypoglycemic episodes report lower general health and greater fear of hypoglycemic events compared with patients who do not experience hypoglycemia. The need to avoid hypoglycemia and the long-term consequences of hyperglycemia remain a challenge in disease management. Patients may prioritize the immediate risk of hypoglycemia over the possibility of future health problems [17]. It has been suggested that patients may intentionally take less diabetes medication (i.e., reduce treatment compliance) or over-eat to increase their blood glucose level in order to avoid hypoglycemia [3,17,18]. Hypoglycemic episodes not only impact the daily clinical management and well-being of patients, but the fear resulting from the side effect may negatively impact long-term diabetes outcomes if acceptable glucose levels are not maintained.

In addition to quantifying the impact of hypoglycemia on patients' HRQoL, these data are important for determining the value of treatment. Health-related utility from the EQ-5D is a generic measure of health status and is often used to determine the relative cost-effectiveness of a given drug or drug regimen [18]. Although these analyses are frequently used to estimate treatment outcomes for patients with type 2 diabetes, few utility measures are available for medication-related side effects that may be important to patients [19]. Although disutility was associated with both the severity and frequency of hypoglycemic symptoms, further research is needed to understand how fear of hypoglycemia impacts health-related utility in patients with type 2 diabetes treated with oral antihyperglycemic agents.

The following limitations need to be considered in the interpretation of the present results. The NHWS sampling frame was stratified by age, gender, and race/ethnicity. Some degree of selection bias in terms of literacy level and socioeconomic status, however, may be present. Individuals without access to internet technology may be under-represented, resulting in a study population with a somewhat higher education and/or income than the national average. Better methods are needed for the classification of hypoglycemia, particularly mild events. Characterization of mild symptoms can be difficult because they may occur due to reasons other than low blood sugar (e.g. headache, hunger, sweating) [20], which may result in over-estimation of hypoglycemia. This may, in part, explain the high overall prevalence of hypoglycemia recorded in this study. Self-reported hypoglycemia is also subject to difficulties of recall. In the present study, patients were asked to report hypoglycemic symptoms that occurred in the previous 6 months. The gold standard for characterization of hypoglycemia requires measurement of blood glucose and documentation of hypoglycemia in the presence of typical symptoms, which may not always be possible for patients [18]. Health-related utility can be affected by many factors. Although the estimates were adjusted for patient characteristics and comorbid disease, there may be other unmeasured factors that influence utility that are unaccounted for. In addition, because of the cross-sectional design of this study, temporal relationships cannot be determined (e.g., the influence of hypoglycemia on subsequent health status, or the influence of lower health status on reporting symptoms of hypoglycemia). Current treatment regimen was comprised of monotherapy, dual therapy, and triple therapy with oral antihyperglycemic agents, which may have confounded individual effect of each agent. Previous medication use, rather than current medications, may also have affected patients' perceptions regarding hypoglycemia.

## Conclusions

In this survey of US patients with type 2 diabetes who were treated with oral antihyperglycemic agents, nearly two-thirds reported experiencing hypoglycemic symptoms. The self-reported hypoglycemic symptoms were independently associated with reduced HRQoL, and the magnitude of this reduction increased with both the severity and frequency of the symptoms. Thus, further research is required to determine if therapeutic agents that have a lower risk of hypoglycemia positively influence both patient quality of life and long-term outcomes.

## List of abbreviations

EQ-5D: EuroQol-5D Questionnaire; HbA_1c_: glycosylated hemoglobin; HFS: Hypoglycemia Fear Survey; HRQoL: health-related quality of life; NHWS: National Health and Wellness Survey.

## Competing interests

The authors are employees of Merck Sharp & Dohme, Corp. (Whitehouse Station, NJ), the sponsor of this study.

## Authors' contributions

EM, QZ, and LR were involved in the concept and design of the study and involved in the data collection and/or analysis. All authors were involved in interpretation of the results and drafting the manuscript. All authors read and approved the final manuscript.
